# Working after cancer diagnosis: should I? shouldn’t I?

**DOI:** 10.3332/ecancer.2014.ed34

**Published:** 2014-04-11

**Authors:** Julie Denning, Nicola Hunter

**Affiliations:** Working Towards Wellbeing, Maynewater Lane, Bury St Edmunds, Suffolk, IP33 2AB, UK

Our story starts with work.

Being at work has been shown to be good for health and improves function and general wellbeing [1]. Following illness and injury, returning to work is an important part of rehabilitation and recovery of normality [4], [17]. Work plays an important role in people’s lives as it provides structure, social support, financial remuneration, a sense of self, a sense of normalcy and a purpose [1]-[3]. Dame Carol Black’s review [4] suggested that work is good for health and that worklessness leads to poorer health. Approximately 6 million people in the UK work while managing a long-term health problem.

Macmillan Cancer Support charity data indicate there are two million people currently living with cancer in the UK, and that this number will double by 2030 [5]. By 2020 almost 1 in 2 people will get cancer in their lifetime and 4 in 10 will not die from it [5]. 2009 figures suggest that 700,000 people of working age have a diagnosis of cancer [6] furthermore, annually, over 109,000 of working age people are diagnosed with cancer [7]. A market survey has indicated that over a 2 year period from 2010 - 2012 there was an increase in income protection cancer claims of 46% [8]. Statistics suggest that people with cancer are 1.37 times more likely to be unemployed than those who have not had cancer [9], yet people surviving cancer want to return to work [10], [11].

As a result of longer life expectancy post-diagnosis, there is an increasing need to provide greater support for people returning to the workplace with cancer and enable them to self-manage. Evidence suggests it is helpful for people to start thinking about a return to work right from the beginning of their cancer journey [11], and that all people working or with potential to work should be offered work support and advice [12].

Unfortunately, vocational rehabilitation (VR) needs are not high on anyone's agenda and few services are available to support people with cancer to return to work. Most rehabilitation services do not address return to work or the associated physical, emotional and practical issues affecting cancer survivors, including fatigue, pain, financial problems and reduced self-confidence. Furthermore, although there is a strong evidence base for VR in other long-term conditions, including musculoskeletal (MSK) and mental health, there is limited evidence for it in cancer [9]. Of the available research, multidisciplinary interventions have been found to be the most effective [9].

According to NCSI [13] research, there are a number of important components of a good VR service. Early intervention is key in order to start the conversation about work and to provide information and support with return to work plans. Work is not often discussed in the clinical pathway, and if it is, it is often downplayed in a ‘think about that later’ approach. Overall there has been found to be a limited provision of information regarding return to work for cancer patients [14] and more worryingly, limited confidence of health professionals to be able to discuss and manage work related issues [15].

What is also critical is having a strong partnership between stakeholder services involved in the person’s care. Links need to be established between treating oncology teams, the GP, line managers and HR departments, Occupational Health departments where they exist, physiotherapists, psychotherapists, case managers (VR and Insurance) and of course the patient. The role of the employer cannot be underplayed; their involvement is critical to the individual’s positive perceptions about work, and importantly return-to-work. Those employers who have kept in light-touch contact with their employee and have provided encouragement and support in phased return-to-work programmes make the path much more straightforward and less stressful for their employee.

Cancer survivorship is more than surgery, chemotherapy and radiotherapy. Access to specialist services that support both physical and psychological elements of survivorship is an important component of Vocational Rehabilitation. Very often patients are discharged from hospital with lingering aches and pains, low self-confidence and worries about whether or not they will return back to their ‘normal’ lives again. Provision of pain management services and support, psychotherapy or coaching, can enable patients to make the difficult transition from patient back to person.

As with the management of all chronic illnesses, a self-management approach needs to be embedded as soon as possible. The philosophy of ‘can do’ is one that can be developed gradually. Case managers can borrow the techniques from occupational psychology and situational leadership [16] to shape and develop an individual’s skill set to transition from dependent patient to independent person. This requires significant skill of the case manager to be able to shape health behaviours so that the person adapts well to their new reality.

In response to the need for a multilevel, multicomponent VR service, Working Towards Wellbeing has designed a cancer-oriented work-support service, delivered by health professionals who specialise in helping people who are living with cancer to stay in work or return to work as soon as possible.

Our service offers early access to a vocational case manager who provides stepped care support and facilitates access to physical and psychological treatment and also to structured exercise programmes. The service flexes around the individual’s needs, and feedback so far has been positive. People like the opportunity to discuss their worries and concerns about cancer, to talk about work, and to have easy access to services that were not provided as an option on discharge from hospital.

We have regular conversations around survivorship recovery, fatigue, chronic pain, fear of recurrence, body image concerns, ‘chemo brain’ and workplace factors. Individuals have felt that it is invaluable to be able to talk to someone separate from their lives. Comments have included: ‘thanks for the chat today, it does help a lot’, ‘It’s lovely to be able to speak to you and to talk things through’, ‘its really cathartic to offload’ ‘Many thanks for an invaluable contribution towards my recovery’.

Our aim is to get a rich stream of outcome data that can inform the development of future VR programmes for patients with cancer and ideally promote the seamless implementation of the service for everyone starting their cancer ‘journey’ to facilitate their recovery and enable improved quality of life in cancer survivorship. We would like to see improved links between the NHS and the workplace to facilitate the transition from a clinical environment towards the working environment for cancer survivors. This is likely to be effective in keeping people at work and improving their function and wellbeing, and decreasing the impact on the NHS in terms of referral and healthcare usage.

So far, we have provided a service which people value and find beneficial both physically and psychologically. Watch this space for our research outcomes. Our story continues.
